# Cases of the First Stage of Cancer and of Fluor Albus

**Published:** 1810-10

**Authors:** J. E. Harrison

**Affiliations:** Thornbury


*Cases of the first Stage of Cancer and of Fluor Albus;* communicated by J. E. HARRISON, of Thornbury.

MRS. Gough, aged between 30 and 40, of a weakly spare habit, the wife of a poor shoemaker, applied to me in the month of July, 1808, being extremely distressed in mind, apprehensive that nothing but the amputation of her breast, which had been four years diseased, could give her a chance of life. I found her much emaciated, with little or no appetite, and hectic; pulse thready, 125, with a large schirrous tumor, of the whole breast, of adamantine hardness, full as large as a child's head nine or ten months old; on the basis there was a livid triangular appearance, somewhat like three claws of a crab, just rising above the surface: I shall call this cancer, or, as the Greeks call it, *καρκινος*. The axillary glands were greatly enlarged and indurated; in such a case, almost hopeless, I thought of Celsus, "Anceps remediam potius quam, nullam," with my own motto, "Nil desperandum:" advising her to trust. I directed an immediate application of as many leeches as she could procure (seven), and a large poultice of folior, cicut. all over the breast, to be renewed twice every twenty-four hours. The next day, July 9th, I directed some cicut. pills with calomel, a grain each, one every night, with a milk diet; and cautioned her to abstain from salted, smoaked, or dried meats. The leeches were generally repeated every week for the first five weeks, with the poultices twice a day, when, in defiance of all my attention, the crab ate himself out, and a large hole was made that discharged a very fœtid ichor. This tumor was the first three years perfectly indolent, of the same colour as the skin, and did not create any pain until the fourth year, nor did it increase very much until then, when it increased rapidly. September, The pills, cicut. poultices, with three grains of opium occasionally, to relieve the lancinating pain, were continued; and acid. mur. applied lighly to the ulcer, now and then, which did not spread much. The latter end of October, several small abscesses burst, that communicated with the first; and as she was extremely weak, with a thready pulse, 120, I directed her to take a corroborating mixture of bark, steel, and extract. cicutæ, cochlear. ij ter. quotidie, correcting her bowels, which frequently threatened a diarrhœa, with repeated small doses of rhubarb. November, The pills, poultices, and bark were continued. December, The pills had touched her mouth and gums; and as I did not wish to bring on a ptyalism, they were discontinued a fortnight. January, 1809, The abscesses began to put on a more favourable appearance. The tumor had subsided more than one half; her pains were mitigated, and the axillary glands almost reduced to the natural size. The same remedies continued. February, The calomel pills were again obliged to be omitted for a fortnight, on account of the soreness of her mouth. Poultices and mixture, with now and then an opium pill, were continued. March, The abscesses begun, slowly, to cicatrize: she was sometimes attacked with violent lancinating pain, occasioned, I believe, by taking cold, which was constantly relieved with opium pills, pro re nata. April, May, June, and July, the same remedies occasionally continued continued, August, September, and October, her appetite mended, she had gathered some strength, and the hectic was cured. Calomel pills frequently omitted, and when taken, only one in three days. Opium pills intirely omitted. Poultices constantly applied. November, only one hole left in her breast, and she herself began to think that there was a possibility of saving her breast and life. Latter end of December, 1809, she was radically cured, and in tolerable good health. Wednesday, August 29, 1810, she has recovered her health and strength, and declares herself to be as well as ever she remembers.

---

JAN. 1, 1806, I was sent for to a very respectable lady, aged 27, mother of one weakly child, eight years old, who had been afflicted many years with fluor albus: it is about eight or ten years since this lady's mother died of a cancer of the uterus; Mrs. ——— had been to London before she applied to me, to one of the most eminent physicians, and was six months under his care without relief: she was much debilitated, had a small thready pulse, 125, hectic heats, little or no appetite, violent pain in the back, loins, and uterine region, with a profuse discharge, which sometimes put on a glairy appearance, at all times fetid, frequent dysury, and never voided urine without pain. Prescribed an emetic twice a week of Pulv. Ipecac. gr. xv. A decoction of marshmallows, with ℥j gum Arabic, dissolved in one pint, for common drink. To live on white, broiled, and roasted meats, and shell fish: to drink at meals a glass or two of old Port wine, with a little brandy and water occasionally: to syringe the vagina with strong green tea, three times a day: to take a decoction of bark with steel, two spoonsful three times a day, to strengthen her, on the days the emetic was not taken. Jan. 15, the remedies continued with gentle exercise. Feb. 20, little alteration for the better. To the emetic half a grain of vitriol. cærul. was added. On the latter end of March the emetics were omitted. The chalybeate mixture, &c. continued. April 7, there was some amendment after the blue vitriol was added to the emetics. May the 9th, the following injection was used ter die. R. Calomel. et extr. cicutæ sing. ʒj. mucilag. gum, Arabic. ℥ss, aq. hordei ℥iv. M. Chalybeate mixture continued, with ʒj extract. cicutæ to ℥viii. Sumend. coch. jss ter quotidie: much better, pulse 86; appetite mended; discharge nearly the the same in color, but less in quantity, menses more or less every fortnight; sometimes mixed with fluor alb. and sometimes not. June 23, although she is upon the whole better, yet the discharge continues alarming, with dysury, pain in the loins, &c. Chalyb. with cicut. &c. continued. July 15, directed this injection to be used ter die. ℞. vitriol. alb. ℈j. Alum Rup. ʒj. Ferr. vitriolat. gr. v. Aq. hordei ℥vj. M. From this day to Oct. 29, perceiving little alteration, excepting the symptoms of hectic were lessened, and the pulse seldom exceeding 86, but the pain in the loins, region of the uterus, and dysury, still continuing, I prescribed the following mixture. ℞. Tinc. Canth. ʒj. Sp. Lavend. C. gutt. xx. Aq. distillat. ℥viij. M. Sumat cochlear, j larg, ter quotidie, and discontinued the chalybeate. Nov. 1, as the Tinc. cantharid. did not affect the bladder, I encreased the dose to ʒij in the ℥viij mixture. Nov. 7, encreased the mixture to ʒiiss. Astringent injection continued. Nov. 11, encreased the Tinc. canthar. to ʒiv. Nov. 29, less discharge, and not so fetid and painful. Dec. 3, as the Tinc. canth. has not affected the bladder, I ventured to increase it to ʒvj in oz. viii mixture, cochlear. j, ter die. Dec. 7. ℞. Tin. cantharid. ℥j. Aq. distillat. ℥vij. M. Cochlear. j, ampl. ter die. This mixture encreased the dysury, brought on a copious purulent discharge with some blood, which was relieved by frequent draughts of decoct. Althææ, and two draughts with Tinc. Opii, gutt. xx in each, immediately, one after the other: in nine hours the pains were arrested. The next day the discharge was lessened considerably, and she was relieved in every respect, but she was alarmed to such a degree, that all my rhetoric could not prevail on her to renew the Tinc. Canthar. in small doses, until the 10th of March, 1807, when she took the following ℞. Tinc. Canth. ʒjv. Aq. distill. ℥viijss. M. Cochlear. j ter quotidie. March 14, mixture continued. March 18, increased Tinc. Canth. to ʒvj. March 19, she was again seized with violent pains, dysury, bloody urine, &c. No relief, with gutt. xl T. Opii in a draught. She fainted several times with agonizing pain; I became *myself* exceedingly alarmed, fearing she would expire under my hands: I directly dipped a double piece of flannel in the following epithem. ℞. Spt. Vin. Camph. ℥iij. Tinc. Opii ʒjss. M. and applied it on the pubis, keeping it continually wet, and gave her a pill of ℞. Camph. gr. iv, Pulv. Opii pur. gr. jss, every hour. Three of the pills, with the epithem, relieved her of her pains, and me of my fears. March 23, she received so much benefit from the last mixture, in every respect, respect, that I prevailed on her to begin again with ʒvj Tinc Canth. in ℥viij of the mixture, as some discharge remained. May 13, as the urinary passage seemed to threaten another dreadful *contest*, I directed one of the following pills every two hours, until the ardor urinæ ceased. R. Camphor. gr. xxiv, Opii pur. gr. viij, Mucil. q. suff. pil. vij, with plentiful draughts of decoct. Althææ, with Gum. Arab. Two of the pills arrested the symptoms with Dec. Alth. May 22, she was in high spirits, pain of the back, dysury, hectic heats, and scarce any discharge remaining, and that without smell, quite bland: I continued the Tinc. Canth. as follows: R. Tinc. Canth. ℥j, Aq. Distill. ℔j. Cochlear. j ter die. June 24, Tinc. Canth. et Dec. Althææ continued. July 10, deemed it adviseable to continue the Tinc. Canth. as the discharge was not totally arrested. Aug. 18, discharge remaining, and her health so much improved, better than it had been for many years, I took leave of her. She bestowed on me many thanks and great praise, &c. which is due *more* to that *ingenious* man, *Dr. Roberton*, than to me: for, if I had not been encouraged by his publication on Cantharides, I should not have persevered with so much resolution.

*N. B.* This lady is a tender, weakly woman. I paid her the utmost attention, and staid with her when she was in those agonizing pains until relief was obtained. It was the worst case I ever met with. I shall say of Drs. Jenner and Roberton, that they are the saviours of millions yet unborn. I have not room for a case of deranged practice, in which ʒij of Calomel were given to an infant of only 15 months old. Of course the child was poisoned, and expired in excruciating pain and misery. Case CROUP.

J. E. H.

*Thornbury, Sept.* 15, 1810.

*P. S.* The menses are now regular every three weeks.

